# Modeling Type 1 Diabetes Using Pluripotent Stem Cell Technology

**DOI:** 10.3389/fendo.2021.635662

**Published:** 2021-04-01

**Authors:** Kriti Joshi, Fergus Cameron, Swasti Tiwari, Stuart I. Mannering, Andrew G. Elefanty, Edouard G. Stanley

**Affiliations:** ^1^ Department of Endocrinology and Metabolism, All India Institute of Medical Sciences Rishikesh, Uttarakhand, India; ^2^ Department of Molecular Medicine & Biotechnology, Sanjay Gandhi Post Graduate Institute of Medical Sciences, Lucknow, India; ^3^ Department of Cell Biology, Murdoch Children’s Research Institute, Parkville, Vic, Australia; ^4^ Department of Endocrinology and Diabetes, The Royal Children’s Hospital, Parkville, Vic, Australia; ^5^ Department of Paediatrics, University of Melbourne, Parkville, Vic, Australia; ^6^ Immunology and Diabetes Unit, St. Vincent’s Institute of Medical Research, Fitzroy, Vic, Australia; ^7^ Department of Anatomy and Developmental Biology, Monash University, Clayton, Vic, Australia

**Keywords:** induced pluripotent stem cells, type 1 diabetes, macrophages, antigen presenting cells, T cells, T cell receptor

## Abstract

Induced pluripotent stem cell (iPSC) technology is increasingly being used to create *in vitro* models of monogenic human disorders. This is possible because, by and large, the phenotypic consequences of such genetic variants are often confined to a specific and known cell type, and the genetic variants themselves can be clearly identified and controlled for using a standardized genetic background. In contrast, complex conditions such as autoimmune Type 1 diabetes (T1D) have a polygenic inheritance and are subject to diverse environmental influences. Moreover, the potential cell types thought to contribute to disease progression are many and varied. Furthermore, as HLA matching is critical for cell-cell interactions in disease pathogenesis, any model that seeks to test the involvement of particular cell types must take this restriction into account. As such, creation of an *in vitro* model of T1D will require a system that is cognizant of genetic background and enables the interaction of cells representing multiple lineages to be examined in the context of the relevant environmental disease triggers. In addition, as many of the lineages critical to the development of T1D cannot be easily generated from iPSCs, such models will likely require combinations of cell types derived from *in vitro* and *in vivo* sources. In this review we imagine what an ideal *in vitro* model of T1D might look like and discuss how the required elements could be feasibly assembled using existing technologies. We also examine recent advances towards this goal and discuss potential uses of this technology in contributing to our understanding of the mechanisms underlying this autoimmune condition.

## Introduction

Type 1 diabetes mellitus (T1D) is an autoimmune disorder disease involving the specific destruction of insulin-producing pancreatic beta cells (1). Beta cell loss leads to primary insulin deficiency and subsequent hyperglycemia, which presents as clinical diabetes. A complex interplay of genetic and environmental factors is thought to trigger beta cell specific autoimmunity. The disease predominantly affects children and young adults and current estimates suggest that more than a million children around the world are affected by T1D, with the prevalence rising by almost 3% each year (2) Our knowledge of how the disorder develops remains imperfect and therefore attempts at preventing or curing the disease have largely not met with success (3).

A major deficit in understanding human T1D has been the lack of appropriate models. While multiple therapeutic interventions have been found effective in the Non obese diabetic (NOD) mouse T1D disease model, none have been translatable to humans (4). This has led investigators to question rodent models as a platform for testing disease therapeutics and has also resulted in a quest for human T1D models (5).

An idealized *in vitro* model of T1D would necessarily enable the incorporation of the large number of variables and cell types that have been implicated in disease development. Indeed, understanding how different cell types and environmental factors interact to contribute to the pathogenesis of T1D will be critical for development of new models.

## Disease Pathogenesis

Current understanding is that T1D is precipitated in genetically susceptible individuals by environmental triggers such as infections, diet, toxins or stress, which initiate the autoimmune response against beta cells. Failure of immune tolerance results in the expansion of autoreactive CD4+ and CD8+ T cells, autoantibody-producing B cells, and activation of the innate immune system, which then collude to lead to beta cell destruction (6, 7).

Most of our understanding of disease pathogenesis has been deduced from rodent models such as the NOD mouse (4). However, emerging data from human biobanks such as the Diabetes Virus Detection (DiViD) study (8) and the JDRF Network for Pancreatic Organ Donors (nPOD) (9) have yielded important details of the human disease pathology and highlighted the differences in rodent and human disease patterns.

The histological hallmark of the disease is the presence of insulitis, i.e., an infiltration of inflammatory cells consisting of T and B lymphocytes and macrophages around and within islets (10, 11). Although variable between subjects, CD8+ T cells have been found to be the predominant immune cell type in the insulitic lesion, followed by CD68+ macrophages, CD4+ T cells and CD20+ B cells (11, 12).

## Overview of Pathogenesis

There are numerous hypotheses regarding the events initiating the processes that eventually lead to T1D. For example, a triggering event, such as a viral infection, may lead to an initial phase of beta cell death causing release of beta cell autoantigens (13). MHC Class I hyper-expression has also been noted on beta cells from T1D tissue samples, potentially making these cells prone to attack from self-reactive cytotoxic CD8+ T cells and further antigen release (14). Islet autoantigens are phagocytosed by antigen presenting cells in the islets, and carried to the draining pancreatic lymph nodes where they are presented to CD4+ and CD8+ T cells (15). Due to loss of central and peripheral tolerance, these self-antigens are recognized by autoreactive CD4+ T cells leading to their activation and proliferation. B cell activation leads to formation of plasma cells and the appearance of autoantibodies against islet proteins (16). These immune cells then infiltrate the islets leading to insulitis and progressive beta cell death (17).

**Figure 1:**
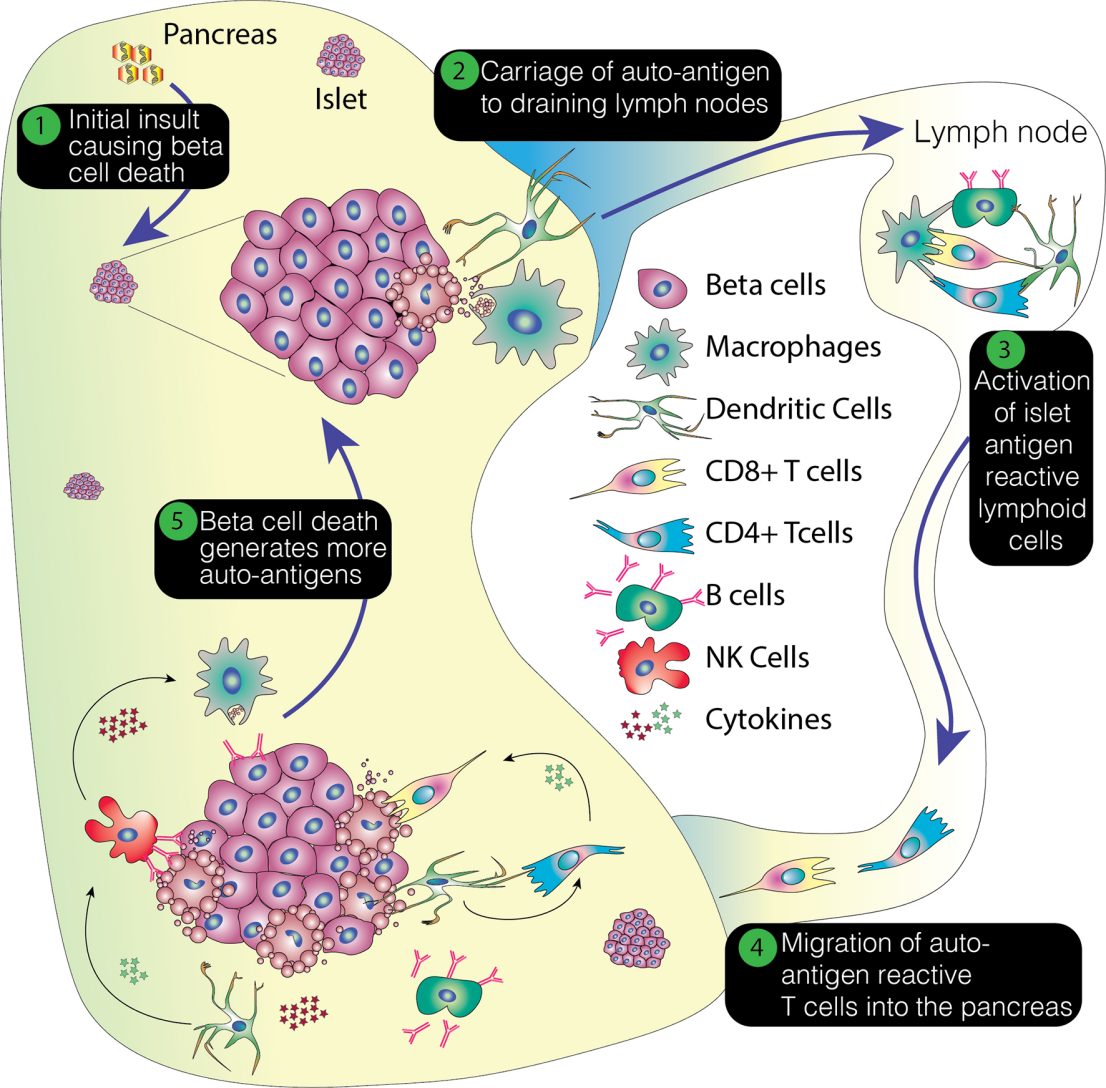
Overview of the pathogenesis of T1D. This process envisages an initial insult that creates beta cell stress or death. The former potentially leading to the production of neoantigens and the latter resulting in the release of beta cell proteins. This damage results in the attraction of immune cells, with emigrant antigen presenting cells picking up and processing the proteins and conveying them to the pancreatic lymph nodes. Here, autoreactive or neoantigen specific T cells are recruited and these then migrate back to the pancreas, potentially promoting further inflammation, stress and cell death. This positive feedback loop ultimately results in the loss of beta cells.

With the initiation of the autoimmune attack, inflammatory cytokines are released which amplify the immune response. These include IL1 and 6, IFNγ, and TNFα. It has been suggested that some of these cytokines directly precipitate beta cell destruction, diminishing insulin secretion from the beta cells and activating cytotoxic T cells (18). They also enhance the expression of HLA class 1 molecules on the beta cells (19). Production of superoxide radicals and high concentrations of nitric oxide increase the damage to the beta cells (18). Inevitably, this damage leads to the further release of beta cell antigens, which may serve to create a feedback loop that reinforces ongoing beta cell destruction. This process is summarized in **Figure 1**.

## The Case for Establishing Human Stem Cell-Based Models of T1D

Studying the disease pathogenesis of human T1D has been challenging for many reasons. The retroperitoneal location of the organ and the inherent risk of pancreatitis make pancreatic biopsies a risky procedure (20, 21), leading to an understandable scarcity of pancreatic tissue samples from affected individuals. The scattered and sparse nature of the insulitic lesions means that multiple tissue samples from one organ are needed for a comprehensive analysis. The tissue itself is difficult to handle due to the high content of pancreatic enzymes, predisposing it to autolysis. In addition to this, T1D has a long pre-symptomatic phase which means that affected individuals only present with established disease where most of the beta cell mass has been lost, making the study of the early disease pathogenesis difficult (5). Therefore, mouse models have been widely used as surrogates of the human disease. However, recent studies on human pancreata have brought to light important differences between human and rodent disease patterns (8, 9, 22).

## Animal Models—The Non-obese Diabetic (NOD) Mouse

NOD mice are prone to spontaneously developing autoimmune diabetes, which mimics many features of human disease such as, islet infiltration by immune cells and the development of autoantibodies (23). However, in distinction to humans, affected mice display an intense insulitis including a peri-insulitic pattern of heavy infiltration, with clusters of lymphocytes often resembling tertiary lymphoid organs. A marked decrease in islet insulin content is seen after week 12 and, after a median period of 18 weeks, diabetes develops in most female mice (22).

## Human Data and Human Biobanks

In recent years, biobanks have been established to collect pancreatic tissue specimens from donors with type 1 diabetes, autoantibody positive individuals, type 2 diabetics and healthy controls. The Diabetes Virus Detection study (DiViD) is one such biobank. DiViD was setup in Norway with the unique premise of collecting pancreatic biopsies from live adult patients newly diagnosed with type 1 diabetes (20). While this study provided valuable information, it also demonstrated the dangers associated with this investigative approach, as serious procedural complications in three out of the six enrolled patients (postoperative leak and bleeding) led to early termination of the study (24). In contrast to the small scale focused DiViD study described above, the Juvenile Diabetes Research Foundation established a multicenter collaborative effort, the Network for Pancreatic Organ Donors with Diabetes (nPOD) in 2007. This tissue biobank supports collection of tissue samples from donors with T1D, autoantibody positive individuals, people with type 2 diabetes (T2DM), and pancreas transplant recipients (with T1D) (25, 26).

*What have human data shown and what are the differences when compared to animal models?*

Analysis of human data suggests that the presence of insulitis in human T1D is much lower than in the NOD mouse with only 10-30% of islets affected on average, however, the variation between patients is large with the DiViD study showing that insulitis varied between 5 and 58% in patients (8, 27). Insulitis was mainly seen to affect the insulin positive islets (33%) with low levels of inflammation found in insulin negative islets (2%) indicating that inflammatory cell influx is predominantly seen in the early phase of the disease with efflux of cells following beta cell destruction and depletion of autoantigen targets (9).

Another important finding from human studies has challenged the classical dogma that >90% of beta cell mass is lost at the time of disease presentation. In fact, it was found that individuals who develop diabetes beyond their teenage years may retain as many as 40% of their insulin positive islets (28), confirming previous knowledge that the disease process is more fulminant in younger cases.

The distribution of insulitis and the degree of immune cell infiltration in human samples is also found to be very different when compared to the NOD mouse (29). While the NOD mouse has been a useful model to study autoimmune diabetes, available human data is bringing to light important differences in pathology between human and rodent disease patterns. It is possible that these differences go some way to explaining why interventions that have been successful in either preventing or reversing the disease process in the NOD mouse have not yielded similar outcomes in human clinical trials (4, 30).

Although studies from human biobanks have gone some way towards filling the gaps in our knowledge, our understanding of the human disease is still incomplete. Issues of limited tissue availability still remain valid, particularly in the current era of enhanced modern diabetes management where death from diabetes related complications is rare. Therefore, there is an urgent need for alternative human models of the disease, which can address species specific aspects of human physiology and allow the study of interventions for disease prevention. This need has therefore paved the way for stem cell derived *in vitro* human disease models.

## Newer Ways of Modeling Disease: Pluripotent Stem Cells

Disease models for T1D are necessary for understanding disease pathogenesis, as a platform for testing potential immune-modulatory therapies and for designing beta cell preservation strategies. *In vivo* studies on immune modulatory therapies have mainly been carried out in NOD mice, and whilst some of these therapies showed success in mice, this success has generally not translated to provide equal efficacy in human disease. This highlights the importance of having species specific disease models, which reflect the complexity and heterogeneity of the human disease process. A human *in vitro* disease model could provide a complementary experimental resource for studying the pathophysiology of the human disease and also for designing potential treatment strategies.

## iPSCs Generation From Human T1D Subjects and Their Use in Studying Disease Pathology

iPSC technology provides an opportunity to generate patient specific cell lines that can be differentiated into tissues of interest and then be used for modeling disease pathology or potentially for cell replacement therapy. iPSCs have been generated from individuals with many different forms of diabetes including T2DM (31), cystic fibrosis related diabetes (32), neonatal diabetes, forms of monogenic diabetes (33, 34), maturity onset diabetes of the young (35–37) and T1D (38).

iPSCs have been successfully used to create human models of diabetes caused by monogenic disorders that effect beta cell development and function such as Wolfram syndrome (33) and insulin gene mutations (34). These experiments not only demonstrated the success of iPSC technology for modeling disease phenotypes but also provided proof of principle data for correcting the disease phenotype.

However, the potential of this system to investigate acquired forms of diabetes has only recently been investigated. T1D is a complex disease to model *in vitro* as the disease has a polygenic inheritance pattern with a heterogenous presentation and a strong influence of environmental factors as potential triggers of autoimmunity. Therefore, simply generating beta cells *in vitro* will likely be insufficient to reproduce the conditions that reflect the *in vivo* disease. Immune cells that have been identified in insulitis lesions from human pancreas, such as CD8+ T cells, CD4+ T cells, macrophages, dendritic cells and B cells (11) would also need to be generated *in vitro* and then co-cultured together with beta cells so as to mimic the pathologic process in the pancreas (39, 40). However, because of the complexity of T cell development, it is unlikely that methods for the generation of autoreactive T cells from iPSCs will be straightforward. As such, it is likely that, in the first instance, autoreactive T cells will need to be obtained from T1D tissue/cell donors. In such a scenario, immune interactions in the disease process could potentially be modeled by recreating key components such as antigen presenting cells and beta cells *in vitro* whilst obtaining autoreactive T-cells from *in vivo* sources.

A number of groups have examined iPSC derived beta cells in the context of T1D tissue donors (38, 41, 42) (**Table 1**). Maehr et al. were one of the first to report the generation of iPSC from individuals with T1D and to differentiate these into beta-like insulin producing cells that were glucose responsive (38). More recently, Millman et al. reported on the generation of iPSCs from three T1D donors and compared their differentiation potential to iPSCs from non-diabetic individuals. Their study showed that beta cells derived from T1D iPSCs were similar to those from non-diabetic individuals in terms of their surface marker expression profile, morphology and* in vitro/in vivo* insulin secretion capacity. They were able to demonstrate that post transplantation into mice, T1D derived iPSC beta cells were equally efficient at rescuing the phenotype of alloxan induced diabetes (42). These experiments reinforce the view that T1D arises from factors that are not intrinsic to the beta cells, whether they are immune cells or environmental triggers. Consistent with this, Millman et al. also examined how the cytokine environment may contribute to beta cell death in the context of type 1 diabetes, hypothesizing that T1D beta cells may be more prone to cytokine induced damage. However, they found that beta cells derived from both diabetic and non-diabetic individuals were equally sensitive to cytokine induced stress, with both showing loss in expression of beta cell markers post exposure to inflammatory cytokines (42). This study therefore further underlines the fact that for studying T1D pathogenesis analyzing islet biology in isolation will not be sufficient and interactions with the immune system will be the key to understanding the complex mechanisms of autoimmune beta cell destruction.

**Table 1:** Generation of iPSCs and relevant cell types from T1D individuals.

Cell type	Starting cell	Results	Reference
Beta like cells	iPSCs generation from fibroblasts of 2 T1D individuals	Insulin & c-peptide positive beta like cells which were glucose responsive	Maehr et al. (38)
Islet like cells	iPSC from skin fibroblasts from 3 T1D individuals and 1-ND	Significant intra-individual variability found with only 1 of 3 iPSC clones from each donor being able to generate INS-positive cells	Thatava et al. (41)
Beta like cells	iPSC from skin fibroblasts from 3 T1D individuals and 3-ND	Generation of C-peptide+/NKX6-1 + glucose responsive beta cells with the ability to ameliorate alloxan induced diabetes in mice. No differences in morphology, marker profile, gene profile, functionality and propensity to cytokine induced stress seen in T1D versus ND iPSC-beta cells.	Millman et al. (42)
Beta like cells	iPSC from peripheral blood from 3 T1D individuals and 1-ND	iPSC beta cells from both, on undergoing ER stress elicit an immune activation response from autologous T cells from both T1D and non-diabetic individuals. T cell activation is specific to beta cells and exposure to iPSC- alpha cells elicits minimal immune activation.	Leite et al. (43)
Macrophages	iPSC from PBMC of 1 T1D individual & 1-ND	iPSC Macs displayed mature morphology and surface marker profile with ability of phagocytosis and capacity to process complex protein mixtures and present relevant epitopes derived from proinsulin C-peptide to TCRs derived from autologous islet infiltrating T cells leading to their activation.	Joshi et al. (44)

T1D, Type 1 Diabetes; ND, Non diabetic; PBMC, Peripheral blood mononuclear cells; ER, endoplasmic reticulum.

A recent study by Hosokawa et al. described the generation of iPSCs derived from patients with fulminant diabetes, a subgroup of the Type 1b non-autoimmune or idiopathic T1D (45). The pathogenesis of this kind of diabetes, which has almost exclusively been reported from Japan, is not well understood. It differs from classical T1D by the rapidity of onset of symptoms, the degree of hyperglycemia and severity of ketoacidosis, almost complete loss of beta cells along with variable alpha cell loss, absence of islet autoantibodies and elevated levels of pancreatic enzymes (46). In distinction to work above related to classical T1D, Hosokawa and colleagues found beta cells generated from fulminant diabetes individuals had an increased sensitivity to proinflammatory cytokine induced damage (45). However since the pathogenesis of this kind of diabetes is believed to be different to classical Type 1A autoimmune diabetes, these results may not be extrapolatable to the classic human T1D disease pathology (46).

In a recent study, Leite et al. simulated T1D relevant immune interactions in an *in vitro* system. They generated iPSC derived beta cells and exposed these cells to ER stress, attempting to replicate the pro-inflammatory islet environment in T1D (**Table 1**). Interestingly, their experiments suggested that stressed iPSC derived beta cells elicit an immune activation response from autologous T cells from both T1D and non-diabetic individuals. They thus concluded that beta cells from T1D individuals are healthy to begin with and the process of islet inflammation makes them stressed and vulnerable to T cell mediated autoimmune destruction (43). This sequence of events is consistent with the hypothesis that an initial assault on beta cells, such as viral infections or an environmental toxin, trigger beta cell damage or stress, which subsequently leads to immune attack and widespread beta cell destruction.

The above experiments have focused on beta cells and their interaction with effector cells of the immune system, particularly CD8+ cytotoxic T cells. However, most current hypotheses regarding the genesis of T1D implicate CD4+ helper cells as underlying drivers of disease because of the very strong genetic association with HLA-class II (47). *In vivo*, the involvement of CD4+ requires the presence of antigen presenting cells: cells that could potentially take up and process antigens from damaged beta cells. For this reason, we investigated the generation of iPSC derived macrophages from individuals with T1D. We were able to demonstrate that these antigen presenting cells had mature functionality and were able to process and present islet lysate and purified synthetic C-peptide to autologous islet infiltrating CD4+ T-cells (44) (**Table 1**). Clearly, the major benefit of the iPSC system is the capacity to exactly match the HLA alleles of antigen presenting cells (APCs) to donor derived T cells. This important feature will be the key advantage of future iPSC-based models of T1D.

## An Idealized Model of T1D

**Figure 2:**
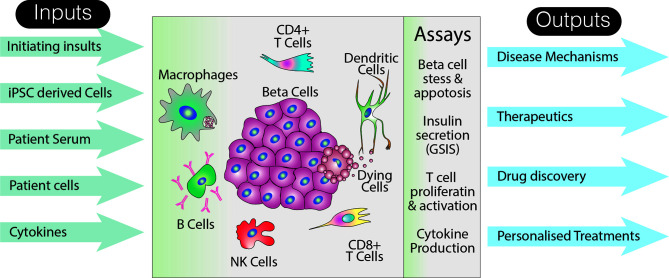
Utility of an idealized model of T1D. The schematic suggests various inputs to the model, how the effects of these inputs could be assayed, as well as long term potential outputs. Note that this representation of the model does not specifically include a host of non-immune and non-endocrine cells which may also impact on the disease pathogenesis. Additionally, although a limited number of assays are shown, analyses specific to particular cell types are likely to increase the breadth and depth of data that could collected from such a system.

The complexity of T1D pathogenesis presents a number of challenges for efforts to create an *in vitro* model that can be used to interrogate the many factors contributing to ongoing islet cell destruction. Key variables that would need to be addressed by a potential stem cell model of T1D include the different cellular insults used to trigger beta cell death or stress, the presence or absence of particular cell types, and, because T cells are an important player, HLA class matching. In order to visualize how such a model could be constructed, it is first necessary to briefly outline what is known about the role that putative environmental triggers and incriminated cell types play in disease onset and progress (**Figure 2**).

### Model Inputs

#### Initiating Insults

##### Genetic Predisposition

T1D has a complex inheritance pattern with genome‐wide association studies identifying more than 60 disease susceptibility loci. The HLA complex has been shown to have the strongest association with T1D and more than half the genetic susceptibility is attributed to this region. Other important loci are the cytotoxic T lymphocyte antigen 4 (CTLA4, IDDM12 locus), PTPN22 (gene encoding lymphoid tyrosine phosphatase) and the IL2RA (interleukin 2 receptor A) locus (48). Most of these loci are associated with immune regulation. Therefore, using iPSCs from T1D individuals carrying high-risk genetic susceptibility alleles would allow the factoring in of underlying genetic factors, which play a role in modulating autoimmunity and mechanisms of impaired immune tolerance in T1D, which lead to disease predisposition.

##### Environmental Factors

There is a complex interplay between genetic disease susceptibility and environmental triggers of autoimmunity. Factors such as viral infections, diet and toxins are believed to be potentiators of islet autoimmunity (49, 50). While an association has been reported with most of these factors, a direct causal relationship has yet to be found. Enteroviruses have been most commonly implicated in disease pathogenesis and are believed to either lead to direct beta cell destruction or to initiate autoimmunity because of molecular mimicry between enteroviral proteins and beta cell antigens (51). Similarly, various dietary factors such as cow’s milk protein, gluten exposure and Vitamin D have long been implicated in the pathogenesis of T1D, however, their roles are still not very well defined. The beta cell stress hypothesis postulates that a combination of any of these factors leads to a state of beta cell endoplasmic reticulum stress promoting generation of neoantigens *via* post-translational modification of islet proteins (52–55).

Any *in vitro* model that seeks to study the entire autoimmune pathogenesis of T1D will likely have to incorporate these triggering factors which link beta cell stress to autoimmunity. It can be envisaged that certain factors such as viruses and their role in beta cell infection and stress could easily be studied in an iPSC derived *in vitro* model of T1D.

#### The Role of Individual Cell Types

##### Beta Cells

Most protocols designed to promote the *in vitro* differentiation of beta-cells from PSCs rely on recapitulating the key developmental steps, which occur during embryogenesis (56). Therefore, an understanding of the steps of early pancreatic organogenesis is essential. Most of the knowledge of developmental biology and signaling pathways involved in pancreatic lineage specification has come from studying rodents (40, 57).

The development of protocols for generating functional endocrine cells has been hampered by a lack of knowledge of pathways involved in final stages of beta cell differentiation. The initial protocols generated cells which though insulin positive, were bi-hormonal and failed to secrete insulin in response to glucose in a glucose-stimulated insulin secretion (GSIS) assay (58–60). However, more recent protocols have described the generation of more mature cells, with development of insulin positive mono-hormonal cells which, show function both *in vitro* and *in vivo*, are glucose responsive, ameliorate diabetes in mice models and are transcriptionally more similar to native beta cells (61–65). Protocol development is an ongoing process, with recent improvements enabling the generation of beta like cells which have a more physiological glucose secretion profile and show appropriate dynamic insulin secretion to high and low glucose challenges (66, 67). However, in spite of these advances, *in vitro* derived beta cells are still ontologically and functionally immature when compared to adult beta cells, with a lower insulin secretion per cell at high glucose, lower glucose stimulation, slower first-phase insulin release and persisting differences in gene expression profiles (68). Studies using single cell RNA- sequencing techniques for transcriptomic profiling of *in vitro* derived beta cells will contribute to a more refined understanding of beta cell maturation pathways and help in development of more evolved beta cell generation protocols (69).

The maturity of the beta cells may indeed be an important factor determining their susceptibility to T cell mediated cell death. Similarly, death induced by cytokines may also be affected by beta cell maturity. It is also conceivable that the degree of functional and transcriptional maturity might also affect the cell’s susceptibility to the initial triggering insult which sets of the process of beta cell death and autoimmunity, potentially a viral insult. With advances in the development beta cell differentiation protocols, generation of mature, functional beta cells that are transcriptionally similar to adult beta cells may become possible. Development of functional adult like beta cells is likely to be an important aspect of *in vitro* models attempting to study T1D *in vitro*.

##### Antigen Presenting Cells

Following an initial insult, macrophages and dendritic cells within the islet and/or draining lymph node are the first responders, phagocytosing cellular debris and processing it for antigen presentation. This is believed the to be the key step in the initiation of autoimmunity and involves the presentation of beta cell autoantigens by professional antigen-presenting cells to CD4+ T cells *via* HLA class II, leading to subsequent T cell activation (15). In studies on pancreata from human subjects with T1D, macrophages have been found to be an important part of the islet infiltrate, thereby underlining their role in the immune events, which precipitate autoimmune diabetes (11, 70). Macrophage depletion and functional inhibition have been shown to reduce the development of autoimmune diabetes in rodent models (71–73). Similarly, NOD mice deficient in CD103+ DCs were found to have a reduced islet infiltration of autoreactive T cells and a corresponding reduction in diabetes incidence (74). Finally, macrophages and DCs may also be involved in causing direct beta cell death by the secretion of proinflammatory cytokines such as interleukin 1-beta (IL1β), tumor necrosis factor alpha (TNFα) and ROS (70, 75). These experiments clearly indicate that antigen presenting cells are likely to play a part in initiating and propagating the T cell mediated autoimmune attack against beta cells.

In an idealized model of T1D, both macrophages and dendritic cells could be generated from iPSCs using established protocols (**Table 2**). In many protocols, PSCs are guided through the sequential stages of hematopoietic development using by stage specific growth factors and cytokines. Induction of mesoderm is achieved by the use of BMP4, activin and FGF2 followed by the addition of VEGF, SCF and FGF to generate hematopoietic precursors. Developing myeloid cells, which are shed from the cultures, are harvested from the supernatant and matured using M-CSF with or without IL3. These cells then pass through an intermediate monocyte stage where CD14+ monocytes can be collected using flow cytometric sorting. Macrophages can then be matured in adherent cultures using high concentrations M-CSF and activated using either Lipopolysaccharide (LPS)/IFNg (classic activation) or IL4 (alternate) activation (86).

**Table 2:** Generation of T1D relevant immune cells from PSCs.

Cell type	Starting cell	Method	Cell functionality and maturity	Reference
Dendritic cells (DCs), monocytes and macrophages	hiPSC-	OP9 mouse stromal cell co-culture for generation of hematopoietic precursors, followed by further differentiation along the macrophage/DC pathway with the use of GM-CSF/M-CSF, Flt3L, SCF and IL1β	DCs capable of cytokine secretion and antigen presentation and activation of allogenic and autologous HLA matched T cells. Macs showed ability of phagocytosis and antitumor activity	Senju et al. (76)
Monocytes and Macrophages	hiPSC-	Embryoid body formation followed by hematopoietic specification by IL-3/M-CSF combination with high dose M-CSF for terminal differentiation	iPSC Macs capable of phagocytosis pro inflammatory cytokine release on LPS stimulation	Lachmann et al. (77)
	hiPSC-	Monolayer culture on a layer of matrigel, an extra- cellular matrix component by using stage specific hematopoietic cytokines to generate monocytes followed by differentiation to macrophages using high dose M-CSF	iPSC Macs showed capacity of bacterial and tumor cell phagocytosis along with relevant cytokine and chemokine release. Phenotypic, functional, and transcriptomic characteristics to peripheral blood monocyte derived macrophage	Cao et al. (78)
	hiPSC-	Embryoid body formation using rotational cultures followed by hematopoietic differentiation using IL-3, M-CSF,GM-CSF and FLT3-L combination with high dose M-CSF for terminal differentiation to mature macrophages. Activation using IFN-γ	iPSC Macs displayed mature morphology and surface marker profile with ability of phagocytosis and capacity to process complex protein mixtures and present relevant epitopes derived from proinsulin C-peptide to TCRs derived from autologous islet infiltrating T cells leading to their activation.	Joshi et al. (44)
Dendritic cells	hiPSC-	Embryoid body formation followed by guided differentiation using hematopoietic growth factors and final DC differentiation using GM-CSF and IL-4	Generation of CD141+ DCs with ability of phagocytosis and features reminiscent of tolerogenic DCs as evinced by capacity of IL-10 secretion, reduced capacity of immunostimulation and polarization of naïve CD4 cells to Tregs.	Sachamitr et al. (79)
	iPSCs derived from an individual with Sjögren’s syndrome	Co-cultured on C3H10T1/2 mouse mesenchymal cells to differentiate into hematopoietic cells	Generation of CD141+ myeloid DCs with ability of phagocytosis. Mature functionality as demonstrated by capacity to stimulate allogenic T cells and present antigen to and activate autoreactive CD4+ T cells.	Iizuka-Koga et al. (80)
Antigen specific T cells	iPSC and hESCs	Directed differentiation and artificial thymic organoids (containing DLL expressing mouse stromal cells). Lentiviral vector introduction of transgenes encoding antigen specific TCRs	Antigen recognition and antigen specific activation CD8+ T cells	Montel-Hagen et al. (81)
	iPSCs from tumor infiltrating CTLs	Mesoderm differentiation followed by co-culture on DLL1 expressing OP9 stromal cells	Antigen recognition and antigen specific activation of CD8+ T cells	Nishimura et al. (82)
	iPSCs from antigen specific lymphocytes	Mesoderm differentiation followed by co-culture on DLL1 expressing OP9 stromal cells	Antigen recognition and antigen specific activation of CD8+ T cells.	Nagano et al. (83)
NK cells	iPSCs from umbilical cord blood	Embryoid body formation, hematopoiesis induction and expansion of NK cells with IL7	*In vitro* and *in vivo* killing of ovarian cancer cell lines.	Hermanson et al. (84)
	iPSCs from peripheral blood	Directed differentiation	Target cell killing and antibody mediated cytotoxicity	Zeng et al. (85)
Antigen specific B cells		N.D		N.D

M-CSF, Macrophage colony stimulating factor; GM-CSF, Granulocyte Macrophage colony stimulating factor; FLT3L, fms like tyrosine kinase 3 receptor ligand; IL-3, Interleukin 3; IL1β, Interleukin 1β; LPS, lipopolysaccharide; IFN-γ, interferon gamma; hESCs, human embryonic stem cells; CTL, cytotoxic T lymphocytes; IL-7, interleukin 7; ND, not done.

*In vitro* PSC derived macrophages have been shown to have similar phenotypic, functional, and transcriptomic characteristics to peripheral blood monocyte derived macrophages (78, 87, 88), suggesting they could be used to provide the antigen processing and presentation functions thought to be key steps in T1D initiation and maintenance. Ideally, iPSCs would be derived from T1D donors from whom islet antigen specific T cells were also available, enabling the interactions between these two cell types to be assessed in a fully autologous HLA setting. However, as noted below, creating or isolating such T cells is likely to be major obstacle to the generation of an authentic *in vitro* model of T1D.

The developmental identity of *in vitro* derived macrophages however remains to be resolved. Most protocols promoting the *in vitro* hematopoietic differentiation of pluripotent stem cells are believed to create cells resembling those generated from embryonic primitive hematopoiesis rather than those derived from adult definitive hematopoiesis. Indeed, there is evidence that PSC-derived macrophages have a primitive embryonic-type macrophage phenotype, predominantly because they expand in the absence of cMyb, a transcription factor required for definitive hematopoiesis (89). However, differences, if any exist, between embryonic and adult origin macrophages in terms of function remains to be elucidated. Moreover, macrophages of fetal origin continue to exist in adult humans in the form of tissue resident macrophages, which self renew with a minimal contribution from adult blood monocytes (90). Therefore *in vitro* derived macrophages may have a role in modeling the functions of these specialized tissue resident macrophages as well (91).

Protocols for the production of dendritic cells from PSCs are limited and most rely on derivation of cells of the myeloid lineage as described above, followed by the further differentiation towards a dendritic cell lineage with the use of GM-CSF and IL4, mirroring methods for deriving DCs from peripheral blood. Most protocols describe an intermediate monocyte stage (92–94) following which blood cells in suspension are harvested and matured in media containing GM-CSF and IL4. These immature DCs then undergo a final maturation step with the use of proinflammatory stimuli like LPS, TNFα (94, 95) or IFNγ, IL1β, PGE2 (79, 93).

Functionally, these DCs have been found to have a cytokine profile, chemotaxis ability and capacity for allogenic T cell stimulation, which is reminiscent of peripheral blood derived myeloid DCs (92, 93, 95). Finally, the antigen presenting functions of iPSC-derived DCs have been used to characterize T cell responses in Sjogrens syndrome, thereby demonstrating that the antigen presenting functions of iPSC derived APCs such as dendritic cells can be used to study the repertoire of pathogenic T cells in autoimmune disorders (80).

##### Lymphocytes in Type 1 Diabetes

T cells are thought to be the primary mediators of beta cell loss in T1D, with cytotoxic CD8+ T lymphocytes mainly responsible for causing beta cell death. The evidence supporting their key role in pathogenesis includes the ability of beta cell specific CD8+ and CD4+ T cell clones to transfer T1D to immunocompromised hosts (96). Furthermore, the use of an anti-CD3 antibody has been shown to reverse T1D in the NOD mouse model (97); a result that has translated to humans with anti-CD3 antibodies shown to preserve beta cell function in recent onset T1D (98) and to delay diabetes progression in secondary prevention trials (99).

##### CD8+ Cytotoxic T Cells

Cytotoxic T lymphocytes (CTLs) are the most common immune cell found in insulitic lesions in human T1D pancreatic specimens (100). They recognize beta cell autoantigens presented by HLA class I expressed on the beta cell surface. CTLs can lead to beta cell death by a variety of mechanisms including the induction of molecules involved in the granule exocytosis pathway such as perforin, granzyme, or granulysin, as well as increased surface expression of death inducing molecules such as Fas ligand and TNF-related apoptosis inducing ligand (101, 102). Autoreactive CD8+ T cells from insulitic lesions in human T1D pancreatic specimens have been found to react to known islet autoantigens such as insulin, islet amyloid polypeptide (IAPP) and islet- specific glucose-6-phosphatase catalytic subunit-related protein (IGRP), pre-proinsulin, GAD65, pre-proislet amyloid protein, and IA-2, providing direct evidence for involvement of these cells in autoimmune beta cell destruction (14).

##### CD4+ Helper T Cells

CD4+ T helper cells are a key player in the pathogenesis of Type 1 diabetes mellitus and have been consistently identified in the inflammatory infiltrate of islets from T1D patients (27). The strong association of HLA class II molecules with the genetic disease susceptibility risk also underscores the important role of CD4+ T cells in the pathogenesis of the disease (48). While CD4+ T cells do not lead to direct beta cell killing, they are important effector cells involved in pro-inflammatory cytokine secretion, which amplify and propagate the immune response and lead to activation of immune cells such as macrophages and CD8+ cytotoxic T cells (102).

Characterizing the antigenic repertoire of these autoreactive T cells has been difficult as the frequency of islet antigen-specific T cells is very low in the most readily available tissue sample, blood, and access to T cells in the islets is limited by the availability of pancreatic tissue samples (103). However, the increasing availability of pancreatic specimens from T1D tissue donors has made possible the study of islet infiltrating autoreactive T cells (104–106). Another approach has been to express T cell receptors (TCRs) from islet infiltrating T cells in an immortalized T cell line to provide a readily available and expandable source of T cells for antigenic testing (107). These studies have played a crucial role in understanding the antigenic targets of CD4+ T cells in T1D, which has not only provided novel insights into disease pathogenesis but may also be useful for testing antigen specific therapies for disease prevention (108).

Any model that seeks to include these cell types needs to be cognizant of the importance of specific T cell receptors that recognize islet antigens. Although a number of methods for making T cells from iPSCs have been published, only a handful of these have addressed the production of T cells bearing specific TCRs (**Table 2**). In short, T cells with specific TCRs can be created by “rejuvenating” T cells isolated from tissue donors or by providing a known TCR in the form of a transgene. In the case of the “rejuvenated” T cells, which can be made from iPSCs generated from T cells, care has to be taken to ensure that the TCR expressed by the iPSC derived T cell is identical to that possessed by the starting cell that was reprogrammed to generate the iPSC. Similarly, iPSC derived T cells expressing a TCR encoded by transgenes may also express endogenously encoded TCRs, potentially complicating the interpretation of antigen specific activation studies.

Although the technical issues surrounding the fidelity of TCRs expressed by *in vitro* derived T cells can be addressed, the work required to generate T cells with specific TCRs from iPSC is still significant. For this reason, direct isolation of T cells from T1D donors may provide a more accessible route for examining this aspect of the autoimmune reactions. However, this path also presents its own challenges, including the phenomenon of T cell exhaustion (82, 109, 110) and the issue of whether the TCR repertoire of T cells in circulation reflects that of autoreactive T cells present with the islets (105, 108). Ideally, an *in vitro* model would incorporate T cells isolated from islets of T1D individuals (44, 104, 107), a scenario that would limit the scope of such a model to deceased tissue donors.

##### B Cells

CD20+ B cell infiltrates have been described in insulitis in human T1D pancreatic specimens (11, 28), however, their exact role in the pathogenesis remains unclear. B cell activation leads to production of autoantibodies against key islet autoantigens that are used as markers for disease onset and as entry points for enrollment in secondary prevention trials (16, 111). However, the conventional understanding is that antibodies by themselves are not pathogenic in T1D (112). There is conflicting data on the need of B cells in initiating autoimmunity as B lymphocyte depletion with the anti CD20 antibody (rituximab) has been associated with reversal of diabetes in the NOD mouse (113) and preservation of beta cell function in newly diagnosed T1D subjects (114). However, in contrast, a report of T1D development in a child with X linked agammaglobulinemia (115) suggests that B cells are not an absolute necessity for disease causation. Nevertheless, B cells can also function as antigen presenting cells and thus may play a role in disease pathogenesis by activating and diversifying the responses of autoreactive T cells in T1D (112).

In an idealized model of T1D, the dual role for B cells as antigen presenting cells and antibody producers could be addressed separately. Specifically, the potential effects of circulating antibodies directed against islet specific antigens could be examined by including patient serum or purified immunoglobulin fractions as input into the *in vitro* model. On the other hand, B cells themselves could be included as APCs. Currently, reports describing protocols for generating B cells from iPSCs have been scant (116, 117) and the robustness of methods for *in vitro* B cell maturation limited. As such, if B cells are to be incorporated into and *in vitro* model of T1D it is likely these will also need to be initially sourced directly from donors. In a similar scenario to that described above for T cells, this approach is likely to exclude the use of B cells producing antibodies with a known specificity.

As a final point, although generation of all the required cells types for a complete model of T1D is onerous, the fact that blood cells can be effectively cryopreserved means that experiments in which cells are recombined into a single culture can be separated from the process of cell generation.

##### Natural Killer Cells

Natural Killer (NK) cells are an innate immune cell that plays a critical role in identifying and killing abnormal cells, particular those that are the target of viral infection or have undergone tumorigenic transformation (reviewed in (118). Historically, NK cells have been classified as lineage negative cells that express CD16 in conjunction with either high or low levels of CD56. The designation “Natural killer” is indicative of this class of lymphocyte’s capacity to kill cells without the requirement for activation by specific HLA antigen complexes, distinguishing them from conventional cytotoxic CD8+ T cells. Indeed, an important characteristic of NK cells is their ability to recognize and kill cells that fail to display self-HLAs, a property important for their role in tumor surveillance. Similarly, NK cells lack of requirement for antigen specific activation means they are first responders to viral infections, recognizing and destroying cells under stress. In addition to the lysis of abnormal cells, another key characteristic of NK cells is their ability to rapidly produce high levels of numerous cytokines and chemokines, putting them in a position to orchestrate immune attacks, as well as serving as an active participate (118).

Only a limited number of studies have examined the role of NK cells in human T1DM (119). Analysis of peripheral blood samples from individuals with T1DM suggested that those with long-standing disease had an NK population that showed reduced levels of activation [for example, reduced production of IFNg (120) and potentially decreased lytic activity (121). Rodacki et al. suggested this reduced NK activity was more likely a consequence than a cause of T1DM, given its association with long-standing disease. Nevertheless, others have suggested that reduced NK activity might make individuals more susceptible viral insults that may precipitate T1DM in the first instance (119)].

Consistent with their role as first responders to viral infection Dotta et al. (122), identified NK cell infiltrates within the islets of 3 T1DM individuals who also showed evidence of Coxsackie B4 enteroviral infection. The presence of NK cells coincided with non-destructive islet inflammation, suggesting these cells could represent a stepping-stone between and initial insult and an expanding inflammatory reaction.

Current protocols for generating NK cells from iPSCs have focused on those representing the myeloid lineages, characterized by expression of CD56 and CD16 (see **Table 2**). These methods have been primarily developed with a view to using iPSC derived NK cells as anti-tumor therapies (84, 85). Given the role of NK cells in detecting cellular stress, inclusion of this cell type in an *in vitro* model of T1DM could provide information related to beta cell stress, whether that be induced by exogenous stimuli or by the presence of the NK cells themselves.

#### Patient Derived Serum—Role of Autoantibodies in T1D

The release of autoantigens following the initiation of autoimmune attack on the pancreatic beta cells leads to the formation of antibodies against key islet proteins such as insulin (micro IAA or mIAA), glutamic acid decarboxylase (GAD), islet antigen 2 (IA-2), and zinc transporter 8 (ZnT8) (13, 123). As noted above the role of autoantibodies in T1D is not well defined. It is believed that the antibodies themselves are not pathogenic and do not cause disease by forming immune complexes as has been described for other autoimmune diseases (124). However, they do predict the risk of development of disease and rate of progression of disease (115). One potential important role played by autoantibodies in the T1D disease process is their effect on autoantigen processing and presentation by class II major histocompatibility complexes (125).

Several effector mechanisms render autoantibodies potentially harmful. These include antibody-dependent, cell-mediated cytotoxicity; release of inflammatory mediators through stimulation of Fc receptors on natural killer cells, macrophages, or mast cells; opsonization of islet autoantigen, which promotes phagocytosis by macrophages; and complement activation with subsequent assembly of the membrane-attack complex (126, 127).

Earlier studies demonstrated that sera from patients with T1D can have a cytotoxic effects on cultured rat beta cells (128, 129). Increased complement activation has been seen in serum of patients with recent onset T1D and similarly was found to cause apoptosis in rat islets (130). A study analyzing human pancreatic tissue specimens from the Network for Pancreatic Organ Donors Diabetes (nPOD) program has also demonstrated evidence of complement activation in the pancreas (131). However, there have been few studies that examine the direct interaction of these antibodies with live beta cells and data on effect of these antibodies on human beta cells is sparse. In the same vein, few experiments have directly addressed the possibility that other serum bound factors may influence beta cell viability or function. An iPSC based model would be a useful platform to study the functional effects of islet autoantibodies/patient serum derived factors on beta cell function and the propagation of the autoimmune process. Indeed, such platforms have been successfully used for modeling autoantibody mediated neuromuscular diseases such as Myasthenia gravis (132).

### Model Outputs

#### Disease Mechanisms

One of the most important uses of this system would be to study disease pathogenesis by *in vitro* assays that could examine beta cell function and immune cell activation

##### Assessment of Beta Cell Mass and Function

- Beta cell apoptosis assays
- Insulin content of beta cells and Glucose stimulated insulin assay (GSIS)

##### Immune Cell Activation

- Cytokine production assays
- T cell activation assays
- T cell proliferation assays

In addition to these cell specific assays, single cell RNAseq analysis could be employed to examine how the complex collections of cells respond to changes in their environment or to the presence of other cell types.

#### Potential Therapeutic Outputs

One application of an *in vitro* immune model of T1D would be to explore interventions that might modify the autoimmune response.

##### Drug Discovery and Screening

With the availability of patient specific iPSCs it is possible to recapitulate disease pathogenesis *in vitro* and to use this knowledge to guide development of patient specific targeted therapies (133).This can be particularly useful in disorders with a long preclinical phase such as T1D where, at the time of clinical disease onset, a significant proportion of tissue function is already lost (134). Disorders for which iPSCs have been used for drug discovery include spinal muscular atrophy (135), familial-dysautonomia (136) and amyotrophic lateral sclerosis (137). The failure of translation of most therapies, which are found successful in rodent models in human trials, has highlighted specific issues that are crucial to consider when designing future intervention trials. These issues include the significant knowledge gaps that exist in the understanding of human disease and the realization that rodent disease patterns and key physiological responses are significantly different from humans. Indeed, emerging knowledge suggests the disease process itself is very heterogenous in humans and therefore personalized strategies for immune intervention may be needed (138). A human iPSC derived *in vitro* model could account for interindividual variations in disease pattern and also circumvent the problems relating to pathophysiological differences between rodent and human disease.

In most autoimmune diseases the therapeutic interventions can be tested even when the disease state is well established. On the contrary, in T1D, intervention strategies would ideally be instituted at the pre-symptomatic phase where significant residual beta cell mass and function still remain. Therefore, iPSC-based models, which recreate the early disease milieu of T1D, are fertile ground for testing strategies for secondary and tertiary preservation in Type 1 Diabetes. Recent trials have focused on the use of immunomodulatory agents which inhibit T cell activation, cytokine action and promote Treg formation such as the use of anti CD3 antibody, CTLA-4 Ig, Anti thymocyte globulin (ATG), anti TNF alpha and IL-2 (139, 140). Cell based therapies such as tolerogenic DCs, Tregs and cord blood cells are also being studied for induction of immune tolerance in T1D. Hematopoietic stem cells have been used to reset the immune system in human trials and trials utilizing mesenchymal stem cells for immunomodulation are also underway (141). An iPSC model would be an ideal testing platform for pre-clinical trials of these therapeutic agents and provide output data relevant to human disease.

Drug repositioning, that is, uncovering new applications for existing drugs, is another application of iPSC technology. This approach has been investigated for conditions such as skeletal dysplasia’s, Alzheimer’s disease and amyotrophic lateral sclerosis (142). Drugs such as hydroxychloroquine (an anti-malarial with immunomodulatory activity) and imatinib mesylate (a tyrosine kinase inhibitor used in chronic myeloid leukemia) are currently being tested in beta cell preservation trials (143) after promising results in preclinical studies. iPSC based models provide a unique human platform for drug discovery and testing of novel therapeutic agents and also for validating the efficacy of these therapeutic agents in pre-clinical studies before translation to clinical trials.

## Potential Limitations

Any model that seeks to recapitulate pathogenic events of human T1D may need to examine all of the cell types that have been implicated in disease causality and progression. At present, this is one limitation of trying to create such a model as robust protocols for generating cells with the correct characteristics are currently not available. As such, rather than trying to incorporate the multitude of environmental, genetic and cellular factors that could potentially affect disease pathogenesis, modeling will need to focus on specific aspects of the disease process that are experimentally tractable. Thus, combinations of iPSC and patient derived native cells will need to be used until more robust protocols for generation of immune cells are available.

In this review, we have not discussed the inclusion of other cell types such as endothelial, epithelial and mesenchymal cells, which may also play a role in disease pathogenesis. Similarly, studies have identified a role for the autonomic nervous system in the control of both insulin and glucagon release, as well as a regulation of islet mass (144). It would be clearly very challenging to construct an islet-like organoid *in vitro* that could fully account for such modulatory neural inputs.

In addition to the non-endocrine cells mentioned above, the endocrine components of the islet itself constitute a complex mixture of multihormonal cell types. Moreover, some of these cell types, such as alpha cells, may have a role in T1D pathogenesis. The *in vivo* islet environment and cross talk between various endocrine cells is believed to be important for normal islet function and hormone release (145). Although many beta cell differentiation protocols generate other islet cell types including alpha cells (59, 60), the relative proportions of cell types generated are often difficult to control. In this respect, the islet-like milieu recreated *in vitro* will, at best, be an approximation to the rich interconnected environment of the native human islet.

Finally, such a model would also find it difficult to take into account the effects of non-islet derived hormones and growth factors within circulation that may collectively contribute to inflammation and beta cell stress.

Our omission of specific cell types and circulating factors is a reminder that any *in vitro* model cannot fully mimic the subtle multi-systemic interactions that occur *in vivo*. Future models which incorporate multi-lineage organoid cultures may circumvent some of these issues. Similarly, our current conception of an *in vitro* model of T1D does not address the initial loss of tolerance, which portends the onset of autoimmunity. Models for examining this question might require improvements in T cell and thymic epithelium differentiation protocols that will allow the study of T cell selection.

## Conclusion

The advent of iPSC technology, which brings the possibility of creating diverse human cell types *in vitro*, has provided the opportunity to construct a fully humanized model of T1D that recapitulates human disease pathology. A major impetus for this work has come from recent improvements in protocols for generating iPSC derived beta cells with mature functionality, allowing investigators to generate the cellular target of autoimmunity in the context of specific HLA haplotypes.

The successful creation of a human iPSC based T1D model will allow a more nuanced understanding of the disease process and help investigators design better beta cell preservation strategies. Such models will also capture the heterogeneity of the human disease process and provide a landscape for testing patient tailored therapies. Finally, and most importantly, stem cell models of T1D will lessen our dependence on rodent disease models and pave the way for better translation of preclinical therapeutic strategies to the clinical arena.

## Author Contributions

KJ reviewed the literature and wrote the paper. FC, ST, SM, AE, and ES were involved with the writing, editing, as well as the key concepts behind the paper. All authors contributed to the article, approved the submitted version, and agree to be accountable for the content of the work.

## Funding

The work quoted in this manuscript was supported by grants from the National Health and Medical Research Council (Australia) (GNT1068866, GNT1079004, GNT1117596, GNT1129861, GNT1138717), the Juvenile Diabetes research foundation (3-SRA-2018-603-M-B), Diabetes Australia (Y19G-STAE), the Government of India: Department of Science and Technology (DST/INT/ISR/P-23/2017), Human resource division (HRD-F.5-6/2013-TS. VII), and from Sanjay Gandhi Post Graduate Institute of Medical Sciences India (A PGI/DIR/RC/844/2018).

## Conflict of Interest

The authors declare that the research was conducted in the absence of any commercial or financial relationships that could be construed as a potential conflict of interest.

## Abbreviations

iPSCs, Induced pluripotent stem cells; T1D, Type 1 Diabetes Mellitus; APC, Antigen presenting cell; TCR, T cell receptor.
